# The roles of Y-box-binding protein (YB)-1 and C-X-C motif chemokine ligand 14 (CXCL14) in the progression of prostate cancer via extracellular-signal-regulated kinase (ERK) signaling

**DOI:** 10.1080/21655979.2021.1993537

**Published:** 2021-12-01

**Authors:** Chen Wang, Na Zhao, Fuyuki Sato, Keiji Tanimoto, Hiroyuki Okada, Yang Liu, Ujjal K. Bhawal

**Affiliations:** a Department of Histology, Nihon University School of Dentistry at Matsudo, Chiba, Japan; b Sichuan Cancer Hospital & Institute, Sichuan Cancer Center, School of Medicine, University of Electronic Science and Technology of China, Chengdu, China; c Pathology Division, Shizuoka Cancer Center, Shizuoka, Japan; d Department of Translational Cancer Research, Research Institute for Radiation Biology and Medicine, Hiroshima University, Hiroshima, Japan; e Department of Pharmacology, Saveetha Dental College, Saveetha Institute of Medical and Technical Sciences, Chennai, India; f Department of Biochemistry and Molecular Biology, Nihon University School of Dentistry at Matsudo, Chiba, Japan

**Keywords:** Prostate cancer, YB-1, CXCL14, EGF, ERK pathway

## Abstract

The cold-shock protein Y-box-binding protein (YB)-1 regulates the expression of various chemokines and their receptors at the transcriptional level. Expression of the orphan chemokine CXCL14 is repressed by EGF induced signaling. The possible links between EGF-mediated YB-1 and CXCL14 as well as the functions of critical kinase pathways in the progression of prostate cancer have remained unexplored. Here we examined the correlation between YB-1 and CXCL14, and the ERK/AKT/mTOR pathways in prostate cancer. Knockdown of YB-1 decreased cyclinD1 expression with an upregulation of cleaved-PARP in human prostate cancer cells. EGF treatment upregulated phospho-YB-1 expression in a time-dependent manner, while treatment with an ERK inhibitor completely silenced its expression in prostate cancer cells. EGF treatment stimulates CyclinD1 and YB-1 phosphorylation in an ERK-dependent pathway. Positive and negative regulation of YB-1 and CXCL14 was observed after EGF treatment in prostate cancer cells, respectively. EGF rescues cell cycle and apoptosis via the AKT and ERK pathways. Furthermore, YB-1 silencing induces G1 arrest and apoptosis, while knockdown of CXCL14 facilitates cell growth and inhibits apoptosis in prostate cancer cells. YB-1 and CXCL14 were inversely correlated in prostate cancer cells and tissues. A significant association between poor overall survival and High YB-1 expression was observed in human prostate cancer patients. In conclusion, our data reveal the functional relationship between YB-1 and CXCL14 in EGF mediated ERK signaling, and YB-1 expression is a significant prognostic marker to predict prostate cancer.

## 1. Introduction

Prostate cancer, which is among the fundamental causes of cancer-linked deaths in the world, is the most prevalent cancer in the human urinary system [1,2]. The mainstay palliative treatment for recurrent or advanced prostate cancer is bilateral orchiectomy or the administration of luteinizing hormone-releasing hormone (LHRH) agonists that work with androgen deprivation therapy [3]. Thus, identifying clinically applicable molecular markers and a better understanding of disease biology will contribute to better survival outcomes for patients with aggressive prostate cancer.

Cold shock proteins like Y-box binding protein 1 (YB-1) are known as multifunctional DNA and RNA binding proteins [4]. YB-1 has been observed to promote notable pro-oncogenic functions in a variety of cancers, such as epithelial and mesenchymal cancers [5], lymphoma and leukemia [6]. YB-1 functions as a prominent prognostic biomarker in cancer and its upregulation is connected to poor patient outcome and drug resistance in a variety of cancers [7–9]. Furthermore, in a mouse xenograft model, it has been suggested that YB-1 promotes prostate tumor progression and androgen ablation, indicating that overexpression of YB-1 may participate in the tumorigenesis and progression of this malignancy [10,11].

Evidence suggests that dysregulation of epidermal growth factor receptor (EGFR) signaling may contribute to cancer cell proliferation, increase tumor vascularization and enhance metastasis [12] and overexpression of EGFR is associated with castration-resistant and high-risk prostate cancer, as well as prostate cancer bone metastasis [13]. Chemokine (C-X-C motif) ligand 14 (CXCL14) functions as a tumor suppressor and is significantly overexpressed in most normal tissues [14]. In head and neck squamous cell carcinoma (HNSCC) cells, EGF has been shown to function as an upstream factor of CXCL14, and the EGFR tyrosine kinase inhibitor can restore CXCL14 [15]. In addition, CXCL14 expression is inversely correlated with EGFR expression in tumor cells [16]. These findings suggest that CXCL14 suppresses tumor progression. Interestingly, it has been clearly demonstrated that YB-1 can bind to EGFR gene promoter elements [17]. Intracellular signaling pathways are involved in mediating various extracellular stimuli via transcription factors such as YB-1 in cancer tumorigenesis and progression including prostate cancer. Previous studies confirmed that AKT mediates the phosphorylation of YB-1 and blocks the mTOR pathway with rapamycin downregulated YB-1 phosphorylation [18]. The phosphoinositide-3-kinase PI3K/AKT/mTOR [19] and MAPK/RSK pathways [20,21] activate YB-1 phosphorylation. Taken together, identifying the role of YB-1 and exploring the correlation between the EGF regulation and relevant kinase pathways in the progression of prostate cancer may clarify the prostate biology as well as aiding the development of novel therapeutics for that disease.

With the hope of being able to aid the development of novel therapeutics, this study aimed at characterizing the EGF regulation in YB-1 and CXCL14 expression and their relevant kinase pathways in prostate cancer cells and tissues. Our present findings using clinicopathological parameters of prostate cancer patients could be instrumental in shedding considerable light on the crucial prognostic role of YB-1 for overall survival in prostate cancer.

## 2. Materials and methods

### 2.1. Cell culture

The DU145 and LNCap human prostate cancer cell lines were purchased from the American Type Culture Collection (ATCC) (Manassas, VA, USA). RPMI-1640 medium (Gibco, Tokyo, Japan) supplemented with streptomycin (100 U/mL), penicillin (100 U/mL), 10% fetal calf serum (FCS) and was used to culture the cell lines. Cells were maintained at 37°C in a 5% CO_2_ atmosphere.

### 2.2. Reagents

Recombinant human EGF (rhEGF) was purchased from PeproTech (Cranbury, NJ, USA). LY294002, PD98059 and Rapamycin were from Calbiochem (San Diego, CA, USA). Cells were treated with rhEGF (10 ng/ml) and LY294002 (10 μM), PD98059 (50 μM) or Rapamycin (10 μM) for various times as noted in the text.

### 2.3 Immunohistochemistry (IHC)

Ninety-one formalin-fixed, paraffin-embedded archived surgical specimens of human prostate cancer were randomly selected for analysis by IHC. Antibodies were used at the following dilutions: 1:50 for YB-1 (Cell Signaling Technology, Danvers, MA, USA); 1:50 for EGFR (Abcam, Cambridge, MA, USA) and 1:50 for CXCL14 (Cell Signaling Technology, Danvers, MA, USA). A Discovery Auto-Stainer was employed to perform IHC according to the automated protocols (Ventana Medical Systems, Inc., Tucson, AZ, USA). The intensities of YB-1, EGFR and CXCL14 were determined by qualitative assessment of two levels: negative and positive. The intensity of YB-1, EGFR and CXCL14 staining was scored as: 0 (no staining), 1 (weak staining), 2 (moderate staining) or 3 (strong staining). The percentages of staining were scored as: 0: 0%, 1: 1–25%, 2: 26–50%, 3: 51–75%, and 4: 76–100%. A YB-1, EGFR and CXCL14 status (score ≤ 4) was defined as negative while scores > 4 represent positive. All proceedings have been conducted in compliance with the Human Genome/Gene Research Ethical Guidelines and were approved by the Ethics Committee of Sichuan Cancer Hospital (Approval Number SCCHEC-03-2018-005).

### 2.4. Small interfering RNA (siRNA) knockdown analysis

Duplexes of siRNAs targeting YB-1 (si03019191), CXCL14 (si00069699) and an AllStars negative control (si0001027281) were purchased from Qiagen (Hilden, Germany). DU145 and LNCap cells were collected after 48 h and 72 h of transfection with RNAiMAX (Thermo Fisher Scientific, Waltham, MA, USA) followed by various analyses as detailed below.

### 2.5. Western blot

RIPA lysis buffer was used to lyse the cultured cells (Santa Cruz Biotechnology, Santa Cruz, CA, USA) and a BCA Protein Assay Kit (Pierce Biotechnology, Rockford, IL, USA) was applied to determine protein concentrations. Antibodies to YB-1 (1:1000, Cell Signaling Technology, Danvers, MA, USA), GAPDH (1:1000, Cell Signaling Technology, Danvers, MA, USA), Cleaved PARP (1:1000, Cell Signaling Technology, Danvers, MA, USA), cyclinD1 (1:1000, Epitomics, Burlingame, CA, USA), ERK (1:1000, Cell Signaling Technology, Danvers, MA, USA), phospho-ERK (1:1000, Cell Signaling Technology, Danvers, MA, USA), phospho-YB-1 (1:1000, Cell Signaling Technology, Danvers, MA, USA), AKT (1:1000, Cell Signaling Technology, Danvers, MA, USA), phospho-AKT (1:1000, Cell Signaling Technology, Danvers, MA, USA), mTOR (1:1000, Cell Signaling Technology, Danvers, MA, USA) and phospho-mTOR (1:1000, Cell Signaling Technology, Danvers, MA, USA) were used. The anti-mouse/rabbit IgG secondary antibodies (1:2000, Cell Signaling Technology, Danvers, MA, USA) were used for 1 h. The bound proteins were visualized using electrochemiluminescence (GE Healthcare, Tokyo, Japan) and were quantitated using a ImageQuant LAS 4000 Mini (GE Healthcare, Tokyo, Japan).

### 2.6. Cell cycle analysis

LNCap cells were treated with LY294002, rhEGF+LY294002, PD98059 or rhEGF+PD98059. After 24 h, the cells were harvested by centrifugation and washed twice with phosphate buffered saline (PBS), and then were fixed in 70% ethanol overnight at 4°C. Cells were then washed with PBS, treated with Propidium Iodide (PI) for 30 min, and analyzed with a BD FACSCalibur system (Beckman Coulter, CA, USA).

### 2.7. Apoptosis assay

An Annexin V-fluorescein isothiocyanate (FITC)-binding assay (BD Biosciences, Franklin Lakes, New Jersey, USA) was used to assess cell apoptosis. LNCap cells were seeded in 6 well plates, were then treated with LY294002, rhEGF+LY294002, PD98059 or rhEGF+PD98059 for 24 h. Cells were washed with PBS and were stained with PI and Annexin V-FITC in each tube for 15 min, after which 1 × Binding Buffer was applied to each tube, analyzed with a BD FACS Calibur system (Beckman Coulter, CA, USA).

### 2.8. qRT-PCR

A RNeasy Mini Kit (Qiagen KK, Tokyo, Japan) was used to extract total RNA [22]. A TURBO DNA-*free*™ Kit (Thermo Fisher Scientific, Waltham, MA, USA) was used to remove contaminating DNA. To examine YB-1 and CXCL14 expression, complementary DNA was obtained by reverse transcription of each total RNA using SuperScript VILO MasterMix (Thermo Fisher Scientific, Waltham, MA, USA). TaqMan probes for YB-1 (Hs00898625_g1), CXCL14 (Hs00171135_m1) and ACTB (Hs99999903_m1) were used for qRT-PCR.

### 2.9. Statistical analysis

Statistical analyses were carried out with GraphPad Prism 7.0 and SPSS 19.0. Statistical significance of differences in gene expression for different clinicopathological parameters was assessed by a two-sided Fishers exact test. Log-rank test and Wilcoxon test were used for overall survival curves. A p value is less than 0.05 were statistically significant.

## 3. Results

### 3.1. Knockdown of YB-1 suppresses cyclinD1 and upregulates cleaved PARP expression

**Figure 1.**
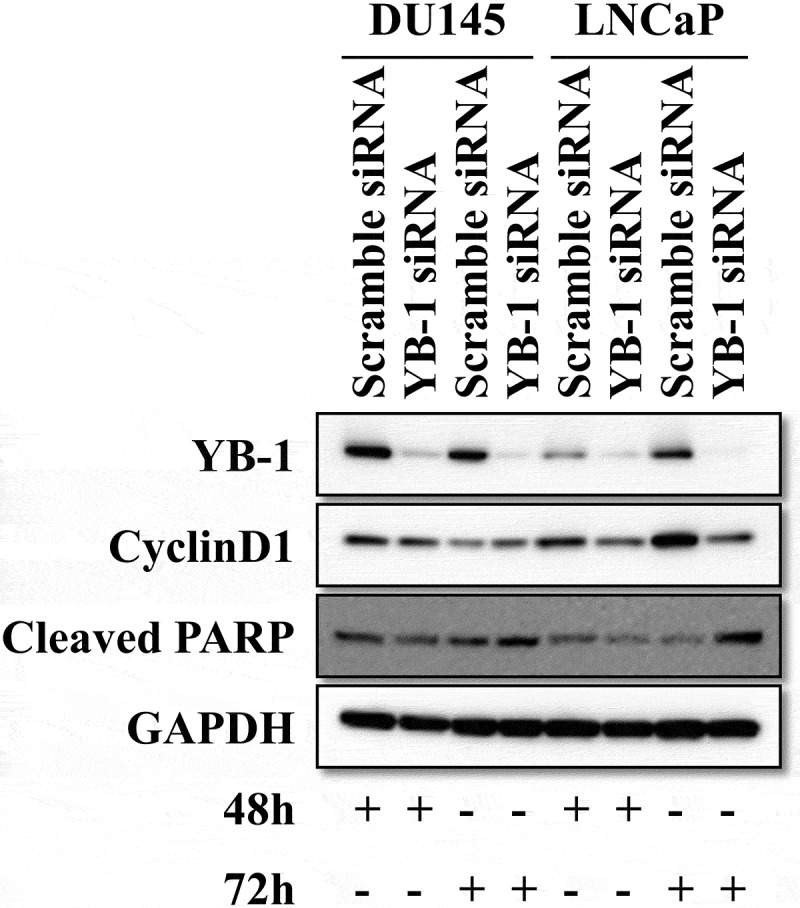
Knockdown of YB-1 suppresses cyclinD1 and upregulates cleaved PARP expression. DU145 and LNCap cells were transfected with a Scramble siRNA or a YB-1 siRNA for 48 h and 72 h. Western blot analysis was performed to assess the expression of YB-1, cyclinD1 and cleaved PARP; GAPDH served as the internal control. A representative image of at least three independent experiments with similar results is shown

To examine the effect of YB-1 on the cell cycle and apoptosis, we performed western blot analyses to determine expression levels of cyclinD1 and cleaved PARP after knockdown of YB-1 for 48 h and 72 h in DU145 and LNCap cell lines. Interestingly, cyclinD1 was significantly downregulated and cleaved PARP was significantly upregulated after YB-1 silencing for 72 h in LNCap cells, while levels of cyclinD1 and cleaved PARP in DU145 cells were only slightly changed at 48 or 72 h after YB-1 knockdown. Therefore, LNCap cells were used for the following experiments (Figure 1).

### 3.2. EGF induces ERK/AKT/mTOR signaling pathways and YB-1 phosphorylation

**Figure 2.**
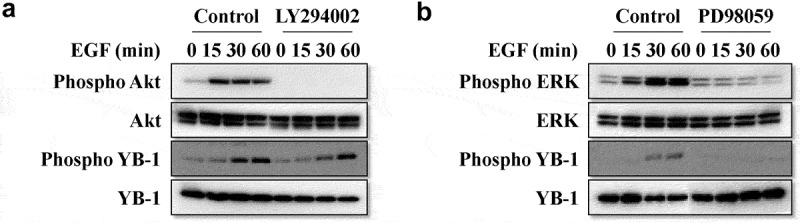
EGF induces the ERK/AKT/mTOR signaling pathways and YB-1 phosphorylation. (a, b) LNCap cells were treated with or without rhEGF and the AKT inhibitor LY294002 or the ERK inhibitor PD98059. The AKT pathway and ERK pathway related proteins, YB-1 and phospho-YB-1 were assessed using WB analysis. A representative image of at least three independent experiments with similar results is shown

**Figure 3.**
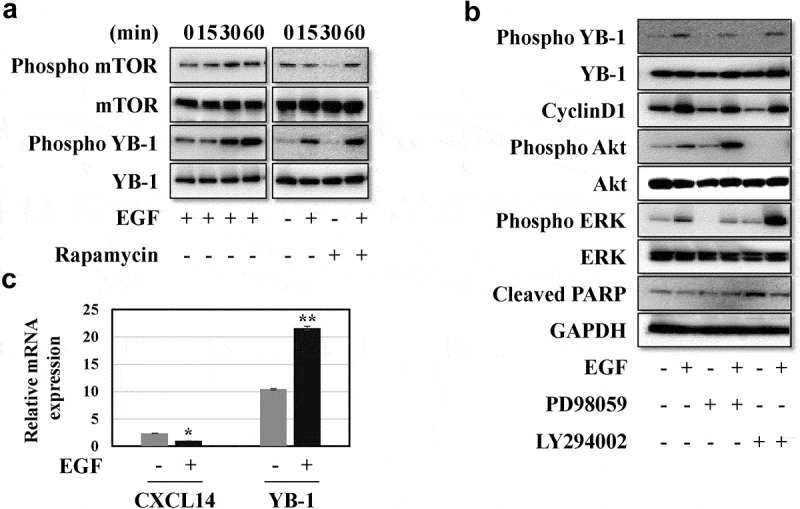
EGF induces the ERK/AKT/mTOR signaling pathways and YB-1 phosphorylation but reduces CXCL14 mRNA expression. (a, b) LNCap cells were treated with or without rhEGF and the AKT inhibitor LY294002, the ERK pathway inhibitor PD98059 or the mTOR pathway inhibitor Rapamycin. AKT-mTOR pathway and ERK pathway related proteins, YB-1, phospho-YB-1, cyclinD1 and cleaved PARP were assessed using WB analysis. (c) CXCL14 and YB-1 mRNA expression levels were measured by qRT-PCR after treatment of LNCap cells with or without rhEGF. A representative image of at least three independent experiments with similar results is shown. Each value represents the mean ± SD (bars) of three independent experiments: *P < 0.05, **P < 0.01

Next, we confirmed the effect of rhEGF on levels of YB-1 and intracellular signaling factors in LNCap cells. Treatment with rhEGF induced the activation of its downstream factors, including AKT, ERK and mTOR in LNCaP cells within 30 min (Figures 2 and 3A). YB-1 was also phosphorylated in rhEGF-treated LNCaP cells. In addition, we used AKT, ERK and mTOR inhibitors to verify the activity of rhEGF to stimulate YB-1 in the regulation of AKT, ERK and mTOR pathways. The EGF-induced YB-1 expression was completely suppressed in PD98059-treated cells but not in LY294002-treated cells (Figures 2B, 3B). This suggests that EGF activates the AKT, ERK and mTOR pathways, and stimulates the phosphorylation of YB-1through the ERK pathway in LNCap cells.

We also characterized levels of the cell cycle related protein cyclinD1 and the cell apoptosis related protein cleaved PARP by western blot analysis. As expected, treatment with LY294002 or PD98059 suppressed cyclinD1 expression while treatment with rhEGF rescued the expression of cyclinD1 when compared to control group (Figure 3b). In contrast, rhEGF treatment slightly downregulated cleaved PARP expression and LY294002 or PD98059 rescued it (Figure 3b).

To identify the link between CXCL14 and YB-1, mRNA expression levels of these genes were identified by qRT-PCR in rhEGF treated LNCap cells. As shown in Figure 3c, rhEGF treated cells had reduced mRNA expression levels of CXCL14, while the mRNA expression level of YB-1 was significantly increased.

### 3.3. EGF rescues cell cycle and apoptosis mediated by blocking of the AKT and ERK pathways

**Figure 4.**
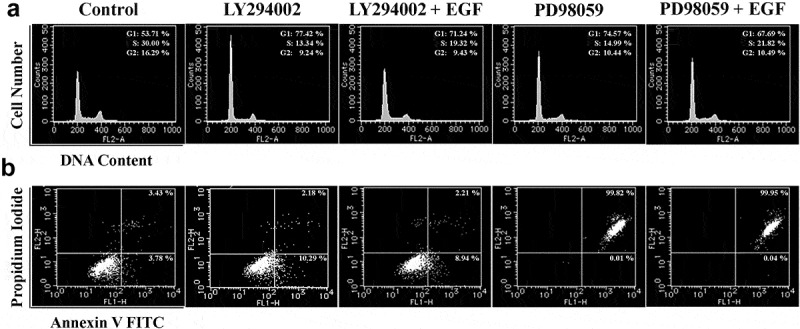
EGF rescues cell cycle and apoptosis mediated by blocking the AKT and ERK pathways. (a) Cell cycle analysis of LNCap cells after treatment with or without rhEGF and the AKT inhibitor LY294002 or the ERK inhibitor PD98059. (b) LNCap cells treated with or without rhEGF and the AKT inhibitor LY294002 or the ERK inhibitor PD98059. The numbers of apoptotic cells were measured by Annexin V-FITC/PI double staining assay

To investigate the effects of AKT and ERK inhibitors on the EGF treated cell cycle distribution and apoptosis of LNCap cells, flow cytometry analyses were carried out. Treatment with EGF facilitated the cell cycle progression and inhibited apoptosis in LY294002 or PD98059 treated cells compared to the control group (Figure 4a, b).

### 3.4. Effects of YB-1 and CXCL14 silencing on cell cycle and apoptosis

**Figure 5.**
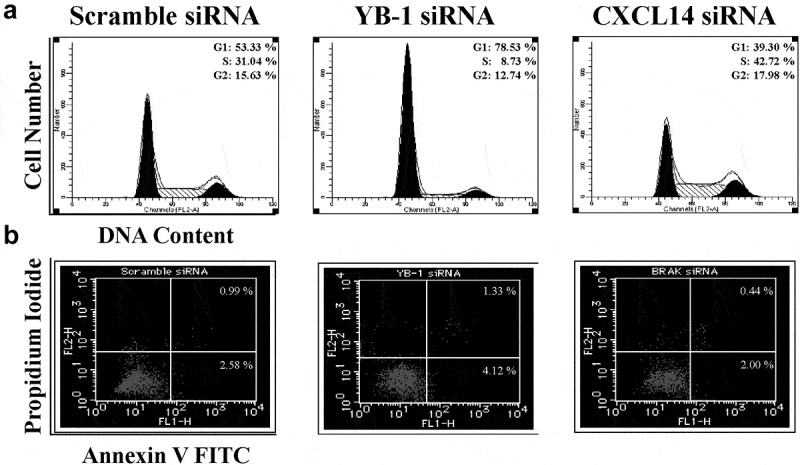
Effects of YB-1 and CXCL14 silencing on cell cycle and apoptosis. (a) LNCAP cells were cultured for 48 h with scrambled siRNA and YB-1 or CXCL14 siRNA, stained with propidium iodide and analyzed for cell cycle by flow cytometry. (b) Cells transfected as in (a) were harvested after 48 h and analyzed for apoptosis by flow cytometry. Percentages correspond to the proportion of cells positive for each profile

Next, we detected the effect of YB-1 and CXCL14 on cell cycle and apoptosis in LNCap cells. YB-1 siRNA group displayed a higher accumulation of cells at G1 phase and increased rates of cell apoptosis (Figure 5). Conversely, CXCL14 siRNA group facilitated cell cycle progression and repressed apoptosis (Figure 5a, b).

### 3.5. CXCL14 expression is inversely correlated with YB-1

**Figure 6.**
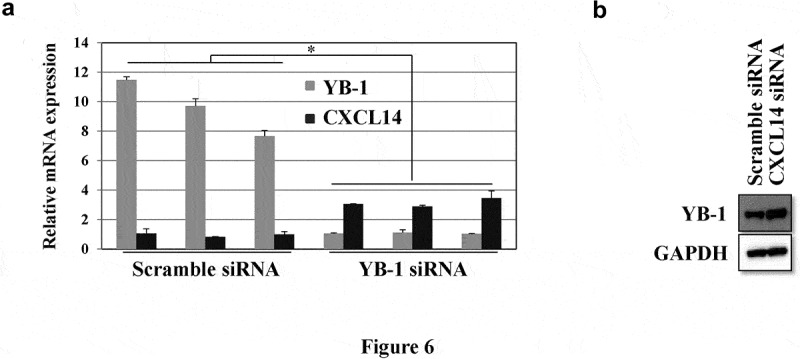
CXCL14 expression is inversely correlated with YB-1. (a) YB-1 and CXCL14 mRNA expression levels were examined by qRT-PCR after knockdown of YB-1. (b) Western blot analysis was performed to assess the expression of YB-1 after knockdown of CXCL14 in LNCap cells; GAPDH served as the internal control. A representative image of at least three independent experiments with similar results is shown. Each value represents the mean ± SD (bars) of three independent experiments: *P < 0.05

To identify the correlation between CXCL14 and YB-1, qRT-PCR and western blot analyses were employed. YB-1 siRNA knockdown induced CXCL14 mRNA expression while silencing CXCL14 significantly upregulated YB-1 protein expression (Figure 6).

### 3.6. Quantification of YB-1, EGFR and CXCL14 expression in prostate cancer tissues

**Figure 7.**
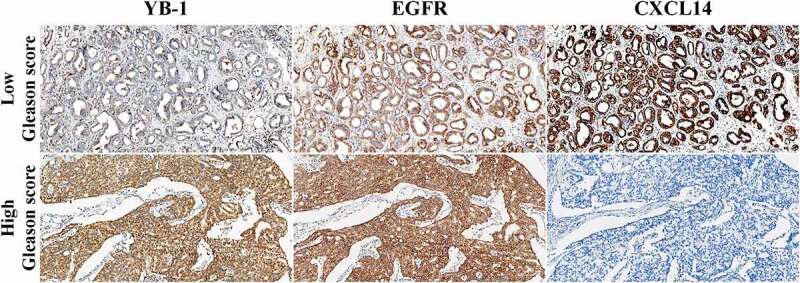
Quantification of YB-1, EGFR and CXCL14 in human prostate cancer tissues. YB-1 was weakly expressed in low Gleason score samples, while CXCL14 and EGFR were positively expressed in the cytoplasm. In high Gleason score samples, the tumor cells were solid without an adenoid structure. YB-1 and EGFR were positively expressed in the nucleus and cytoplasm, respectively, while CXCL14 was negatively expressed in these tissues

Next, we examined the association of EGFR and YB-1 as well as CXCL14 expression with clinical and pathological characteristics in human prostate cancer tissues using IHC. In low Gleason score prostate cancer samples, tumors were nodular and expanded with well-defined boundaries. The glands were well differentiated, rounded or elliptic in contour, without angular fusion. Representative IHC images showed that YB-1 was weakly expressed in low Gleason score samples, while CXCL14 and EGFR were positively expressed in the cytoplasm (Figure 7). In high Gleason score samples, the tumor cells were solid and without adenoid structure. YB-1 and EGFR were positively expressed in the nucleus and cytoplasm, respectively, while CXCL14 was negatively expressed in high Gleason score samples (Figure 7).

### 3.7. Protein expression levels of YB-1, EGFR and CXCL14 correlated with overall survival and clinicopathological parameters in human prostate cancer patients

**Figure 8.**
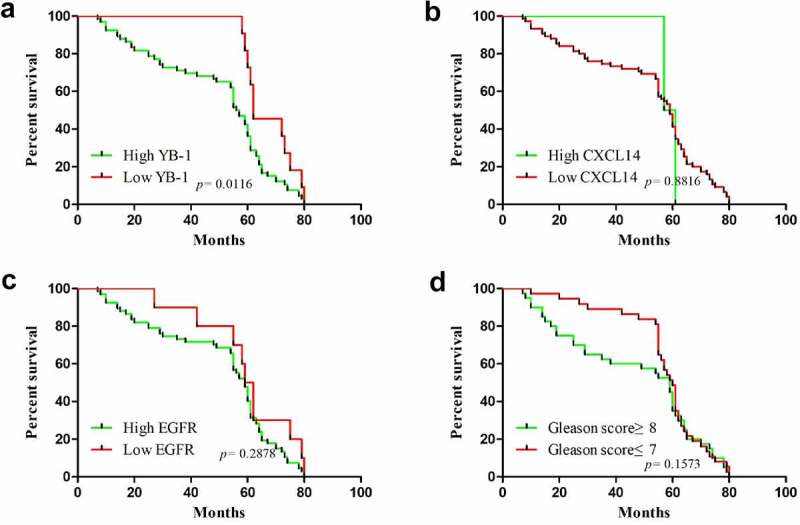
Correlation between overall survival of prostate cancer patients in YB-1, EGFR and CXCL14 expressing tissues. (a) The overall survival of prostate cancer patients with a high expression level of YB-1 was much lower than that of patients with a low level of YB-1(P = 0.0116); (b-d) There was no significant difference in the overall survival of prostate cancer patients with EGFR or CXCL14 expression levels or with Gleason score

The correlation between the mRNA expression levels of YB-1, EGFR and CXCL14 and the clinicopathological characteristics in human prostate cancer is summarized in Table 1. Expression of YB-1 correlated significantly with Gleason grade (p < 0.0001), histological grade (p = 0.001), lymph node metastasis (p = 0.009) and bone metastasis (p = 0.015). EGFR expression correlated significantly with Gleason grade (p < 0.0001), histological grade (p = 0.001) and bone metastasis (p = 0.015). No significant correlation was observed between CXCL14 expression and any of the clinicopathological parameters. Next, we analyzed associations between the survival time of prostate cancer patients and levels of YB-1, EGFR and CXCL14 expression. As shown in Figure 8a, the overall survival of prostate cancer patients with a high level of YB-1 was significantly lower than patients with a low level of YB-1 (*p* = 0.0116). While the overall survival of EGFR and CXCL14 expression and the Gleason score of prostate cancer patients is without significant difference (Figures 8B–8D).

**Table 1.** Correlation of YB-1, EGFR and CXCL14 expression with clinicopathological features in prostate cancer cases

		YB-1 expression			EGFR expression			CXCL14 expression		
Clinicopathological factors	N	≤4	>4	P-value	≤4	>4	P-value	≤4	>4	P-value
Total	91	11	80		11	80		89	2	
**Age (years)**				**0.654**			**0.635**			**1.000**
<61	11	2	9		2	9		11	0	
≥61	80	11	69		10	70		78	2	
**Gleason grade**				**< 0.0001**			**< 0.0001**			**0.200**
≤7	41	11	30		11	30		39	2	
≥8	50	0	50		0	50		50	0	
**Grade**				**0.001**			**0.001**			**1.000**
Well and moderate (≤2)	30	9	21		9	21		29	1	
Poor (≥3)	61	2	59		2	59		60	1	
**Lymph node metastasis**				**0.009**			**0.530**			**1.000**
Yes	43	1	42		4	39		42	1	
No	48	10	38		7	41		47	1	
**Bone metastasis**				**0.015**			**0.015**			**0.538**
Yes	29	0	29		0	29		28	1	
No	62	11	51		11	51		61	1

## 4. Discussion

YB-1 is a versatile protein that is constitutively involved in the regulation of prostate cancer cell biology. This study unequivocally emphasized the prognostic importance of YB-1 in prostate cancer progression. We suggest that silencing of YB-1 would exacerbate cell growth, ameliorate apoptosis, and consequently result in improved overall survival of prostate cancer patients. Our data suggests that a new diagnostic and therapeutic approach for managing prostate cancer patients is primarily related to the changes of EGF regulation in YB-1/ERK axis.

Numerous studies have shown that YB-1 may act as a potential indicator to predict drug resistance and poor prognosis in a variety of cancers [7–9,23–25]. In previous studies, YB-1 is coordinately expressed with EGFR in primary human breast cancer and cervical cancer specimens, and the expression of EGFR is dependent on functional YB-1 [26–28]. In addition, YB-1 is a target of growth factors numerous cancers [29]. Also, YB-1 has been implicated in promoting cancer proliferation via activating EGFR signaling in an exogenous EGF-independent manner [17]. Consistent with those previous findings, our present study proved that an EGF-dependent increase of phospho ERK and phospho YB-1 could modulate the progression of prostate cancer. The significant correlation in the Gleason score, histological grade and bone metastasis resulting from IHC analysis in prostate cancer patients could lead to the fundamental significance of YB-1 in prostate biology. YB-1 is reported to be activated by the Ras/ERK pathway. ERK pathway target genes have also been identified to mediate the expression level of YB-1 in colorectal cancer [21]. Moreover, YB-1 expression is regulated by ERK inflammatory conditions and the ERK signaling pathway has been implicated in YB-1 induced rat mesangial cell proliferation [30]. We also found that EGF induced the ERK pathway and activated YB-1 phosphorylation in LNCap cells. LY294002 or PD98059 suppressed the expression of phospho-YB-1 while EGF treatment rescued that expression. The present study also demonstrated that the EGF-induced expression of phospho-YB-1 was partially blocked by the mTOR inhibitor rapamycin and by the AKT inhibitor LY294002, indicating that AKT-mTOR may regulate YB-1 phosphorylation in LNCap cells. AKT regulates many cellular functions, including growth, proliferation, and survival. A previous study suggested that both EGFR and AKT are upstream signaling activators of YB-1 [18] and are essential for controlling YB-1 activation in breast cancer [31,32]. In melanoma cells, the MAPK and PI3K/AKT signaling pathways activate and increase YB-1 phosphorylation [11]. It has also been reported that phospho-AKT binds to and phosphorylates YB-1 while mTOR does not phosphorylate YB-1 protein in breast cancer [18]. In line with those reports, our study revealed that the mTOR pathway partially regulates YB-1 phosphorylation.

An increasing number of studies has shown that ERK activates YB-1 phosphorylation [33], that AKT mediates YB-1 phosphorylation and that blocking mTOR with rapamycin also suppresses YB-1 phosphorylation [18]. An ERK inhibitor comprehensively suppressed YB-1 phosphorylation and cyclinD1 expression, while it induced cleaved PARP expression. A similar result was obtained with YB-1 silencing in prostate cancer cells, which suggests that YB-1 regulates cyclinD1 and/or cleaved PARP expression via the ERK pathway in prostate cancer. This is in line with published reports that ERK pathways might be implicated in YB-1 signaling [19–21]. In addition, our immunohistochemical analysis along with western blot and cell cycle data indicate the involvement of ERK in prostate cancer progression.

Upregulation of YB-1 expression was related to a high Gleason score in human prostate cancers [10]. Like those results, we also showed thatYB-1 expressionwas linked to the Gleason score. Our IHC study illustrated that YB-1 and EGFR are significantly expressed in prostate cancer tissues with a high Gleason score, while CXCL14 was lowly expressed (Figure 7, Table 1). Strong expression of YB-1 and EGFR are closely related to the Gleason score, histological grade, and bone metastasis, suggesting that YB-1 and EGFR may be important factors that promote the progression of prostate cancer. We found high YB-1 expression had a shorter survival time in patients with prostate cancer than those exhibiting low expression. Our results showed YB-1 expression may result in the poor clinical outcome of patients with prostate cancer and may be a potential cancer marker for this tumor.

The tumor suppressive or tumor supportive roles of CXCL14 in cancer depends on the specific type of tumor [34–37]. Moreover, the CXCL14 gene was selected as a benchmark to evaluate the risk of recurrence in breast cancer [38]. In addition, CXCL14 correlates with poor patient outcome in oral cancers and has significant effects on the tumorigenesis and proliferation of oral cancers by mediating DNA methylation and leukocyte migration [16]. Zeng et al. analyzed CXCL14 protein expression and suggested it is abundantly expressed in colorectal tumor tissue [39]. This clearly implies that CXCL14 is associated with disease recurrence and poor prognosis and is relevant to tumor-node metastasis (TNM) stage, differentiation grade and tumor size. Instead, the associations between increasing expression levels of CXCL14 protein and better patient outcomes have been verified in breast and colorectal cancers [40,41]. Here, our IHC data revealed a low-level expression of CXCL14 and a high EGFR expression in prostate cancer tissues. A high CXCL14 expression exhibited a longer survival time in prostate cancer patients. A previous study demonstrated that EGF is an upstream factor of CXCL14, and that an EGFR tyrosine kinase inhibitor can restore it in HNSCC cells [15]. We observed high expression levels of EGFR in human prostate cancer tissues and a low or minimal EGFR expression had the ability to prolong overall survival. Our study also showed that EGF regulate CXCL14 transcription, and that CXCL14 is inversely correlated with YB-1 in prostate cancer cells (Figure 3c and Figure 6), suggesting a possible mechanism of YB-1 regulation in the cell cycle and in apoptosis in prostate cancer. The fundamental regulatory mechanism of YB-1 and CXCL14 in prostate cancer needs to be further explored.

Collectively, our findings demonstrated that YB-1 expression was substantially related to prognosis of prostate cancer patients. YB-1 regulated cyclinD1 and cleaved PARP via the ERK pathway in prostate cancer. CXCL14, on the other hand, was downregulated in prostate cancer, and was negatively associated with YB-1, thus it may be a valuable prognostic role for predicting future disease states and reflecting cancer cell aggressiveness.

## 5. Conclusion

In conclusion, our results suggested that EGF regulates YB-1 and CXCL14 via ERK signaling. YB-1 and CXCL14 were inversely correlated in prostate cancer. Furthermore, we provide clues to examine YB-1 as an imperative prognostic biomarker for predicting prostate cancer survival. Additional research is underway to ascertain functional regulation of YB-1 in prostate cancer. The present study contributes to understanding the functional mechanism in the prostate cancer progression and should promote the development of novel therapeutics and provide new gene targets for future studies.

## Data Availability

The data that support the findings of this study are openly available in figshare at https://doi.org/10.6084/m9.figshare.16903885.v1

## Abbreviation

Y-box–binding protein (YB-1); Chemokine (C-X-C motif) ligand 14 (CXCL14); Extracellular-signal-regulated kinase (ERK); Epidermal growth factor receptor (EGFR); Head and neck squamous cell carcinoma (HNSCC); Recombinant human EGF (rhEGF); Immunohistochemistry (IHC); Small interfering RNA (siRNA); Phosphate buffered saline (PBS); Propidium Iodide (PI); Fluorescein Isothiocyanate (FITC); insulin-like growth factor 1 (IGF-1); Tumor-node metastasis (TNM)

## Research highlights

1. EGF signaling regulates expression of YB-1 and CXCL14 in prostate cancer.
2. YB-1 and CXCL14 are inversely correlated in prostate cancer.
3. YB-1 regulates cyclinD1 and cleaved-PARP via the ERK pathway in prostate cancer.
